# Ticks and associated pathogens collected from dogs and cats in Belgium

**DOI:** 10.1186/1756-3305-6-183

**Published:** 2013-06-19

**Authors:** Edwin Claerebout, Bertrand Losson, Christel Cochez, Stijn Casaert, Anne-Catherine Dalemans, Ann De Cat, Maxime Madder, Claude Saegerman, Paul Heyman, Laetitia Lempereur

**Affiliations:** 1 Laboratory of Parasitology, Faculty of Veterinary Medicine, Ghent University, Merelbeke, Belgium; 2 Laboratory of Parasitology and Pathology of Parasitic Diseases, University of Liège, Liège, Belgium; 3 Research Laboratory for Vector Borne Diseases, Queen Astrid Military Hospital, Brussels, Belgium; 4 Bayer Health Care, Animal Health Division, Diegem, Belgium; 5 Department of Animal Health Institute of Tropical Medicine, Antwerp, Belgium; 6 Department of Veterinary Tropical Diseases, Faculty of Veterinary Science, University of Pretoria, Private Bag X04, Onderstepoort, Pretoria 0110, South Africa; 7 Research Unit of Epidemiology and Risk Analysis Applied to the Veterinary Sciences, University of Liège, Liege, Belgium

**Keywords:** Ticks, Dermacentor reticulatus, Dogs, Cats, Belgium, Borrelia, Anaplasma, Rickettsia

## Abstract

**Background:**

Although *Ixodes* spp. are the most common ticks in North-Western Europe, recent reports indicated an expanding geographical distribution of *Dermacentor reticulatus* in Western Europe. Recently, the establishment of a *D. reticulatus* population in Belgium was described. *D. reticulatus* is an important vector of canine and equine babesiosis and can transmit several *Rickettsia* species, *Coxiella burnetii* and tick-borne encephalitis virus (TBEV), whilst *Ixodes* spp. are vectors of pathogens causing babesiosis, borreliosis, anaplasmosis, rickettsiosis and TBEV.

**Methods:**

A survey was conducted in 2008-2009 to investigate the presence of different tick species and associated pathogens on dogs and cats in Belgium. Ticks were collected from dogs and cats in 75 veterinary practices, selected by stratified randomization. All collected ticks were morphologically determined and analysed for the presence of *Babesia* spp*., Borrelia* spp., *Anaplasma phagocytophilum* and *Rickettsia* DNA.

**Results:**

In total 2373 ticks were collected from 647 dogs and 506 cats. *Ixodes ricinus* (76.4%) and *I. hexagonus* (22.6%) were the predominant species. *Rhipicephalus sanguineus* (0.3%) and *D. reticulatus* (0.8%) were found in low numbers on dogs only. All dogs infested with *R. sanguineus* had a recent travel history, but *D. reticulatus* were collected from a dog without a history of travelling abroad. Of the collected *Ixodes* ticks, 19.5% were positive for *A. phagocytophilum* and 10.1% for *Borrelia* spp. (*B. afzelii, B. garinii*, *B. burgdorferi s.s., B. lusitaniae, B. valaisiana* and *B. spielmanii)*. *Rickettsia helvetica* was found in 14.1% of *Ixodes* ticks. All *Dermacentor* ticks were negative for all the investigated pathogens, but one *R. sanguineus* tick was positive for *Rickettsia massiliae.*

**Conclusion:**

*D. reticulatus* was confirmed to be present as an indigenous parasite in Belgium. *B. lusitaniae* and *R. helvetica* were detected in ticks in Belgium for the first time.

## Background

The most common tick in North-Western Europe is the sheep or castor bean tick, *Ixodes ricinus.* This tick species is also widely distributed in Belgium [1]. *Ixodes* ticks are vectors of a broad range of pathogens of medical and veterinary importance, such as *Babesia* spp., *Borrelia* spp., *Anaplasma phagocytophilum*, *Rickettsia* spp., *Bartonella* spp. and tick-borne encephalitis virus (TBEV). The brown dog tick, *Rhipicephalus sanguineus* and the European meadow tick, *Dermacentor reticulatus* are occasionally introduced into Northern Europe with imported dogs or by dogs travelling from endemic areas. *R. sanguineus* is a vector of, among others, *Babesia vogeli, Ehrlichia canis, Hepatozoon canis, Rickettsia conorii* and *Cercopithifilaria* spp. *D. reticulatus* is an important vector of canine and equine babesiosis and can transmit *Rickettsia* spp., *Francisella tularensis*, *Coxiella burnetii* and TBEV. Until recently, the French-Belgian border was considered to be the northern boundary of the distribution of *D. reticulatus* in Western Europe [2]. However, recent reports indicated that the habitat of *D. reticulatus* is expanding in North-Western Europe, with several foci now present in The Netherlands [3] and in Germany [4]. The occurrence of autochthonous cases of canine babesiosis in Belgium during the last two decades [5] suggests that this tick species could also be indigenous in this country. Low numbers of *D. reticulatus* were previously found on dogs in Belgium (Losson *et al*., personal communications), but it was not clear whether these ticks belonged to indigenous tick populations or were imported after travelling abroad and the presence of tick-borne pathogens in these ticks was not investigated. Therefore, a new survey was conducted in 2008-2009 to investigate the presence of different tick species collected from dogs and cats in Belgium and their associated pathogens. Dogs and cats were chosen, because several tick-borne diseases are of clinical importance in dogs and/or cats (e.g. babesiosis and ehrlichiosis) and because dogs and cats live in the close vicinity of their owners and can act as direct sentinels for infection of humans [6]. The approach of gathering data on the distribution of (zoonotic) vector-borne diseases through a veterinary survey is consistent with the ‘One Health’ concept [7,8]. Based on the recurrent collection of *D. reticulatus* ticks from a dog in this study, the presence of an indigenous *D. reticulatus* population in Belgium was confirmed by flagging at the study site [9].

## Methods

### Tick collection

**Figure 1:**
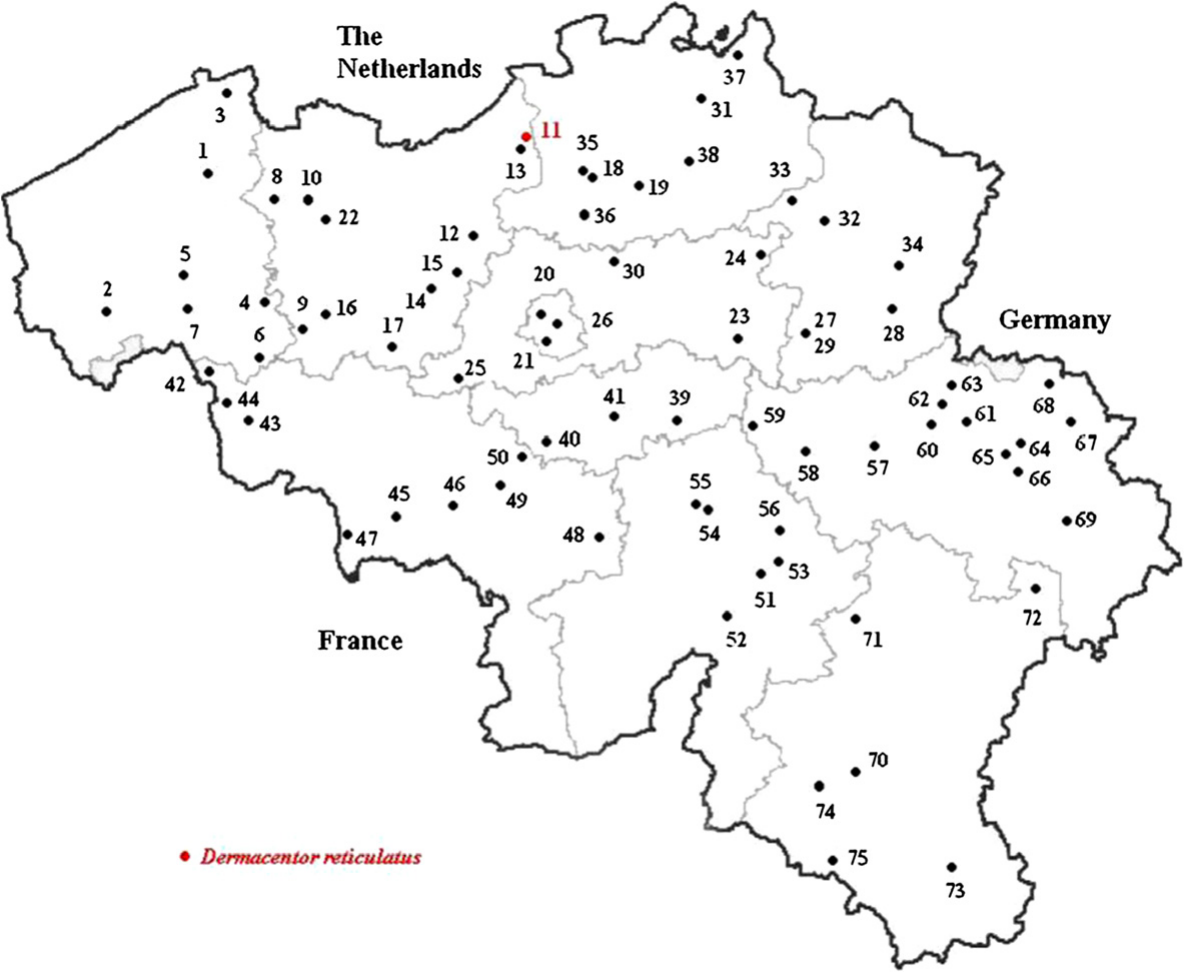
**Distribution of selected veterinary practices and presence of an indigenous *Dermacentor reticulatus *population (red dot) in Belgium.**

A nationwide survey was performed in Belgium from April 2008 to April 2009. For each of the 25 veterinary districts, 3 companion animal practices were selected by stratified randomization (Figure 1). Ticks from cats and dogs, randomly submitted to seventy-five veterinary practices, were collected and preserved in 80% alcohol. For each animal enrolled in the study, information was recorded by the veterinarian about location, date of collection, animal description (dog/cat, age, sex, breed), the location on the host where the tick was found, whether the animal had travelled outside Belgium during the previous 2 weeks and whether the animal showed clinical signs that could be associated with a tick-borne disease, such as fever, apathy, anorexia, arthritis, anaemia or meningitis. The ticks were identified to species level, with stage and sex recorded, using a standard morphological key [10]. When *D. reticulatus* ticks were found on an animal without history of travel to a country known to be endemic for this tick species, the veterinarian or the owner was contacted for more information regarding the area in Belgium where the animal might have acquired the tick. These areas were subsequently surveyed for the presence of *D. reticulatus* by flagging the vegetation with a flannel cloth [9].

### DNA extraction, PCR amplification and sequencing

One tick (nymph or adult) per animal was selected for DNA analyses, but when several tick species were present on a given animal, one tick of each species was randomly selected for further analysis. Tick DNA extraction was performed using a protocol with proteinase K [11]. In order to detect false negative results due to PCR inhibition and to validate the efficiency of the DNA extraction, a PCR targeting a 325 bp DNA fragment corresponding to the tick 16S rRNA gene was included, using 16S + 1 and 16S-2 primers [12].

The presence of *Babesia* spp., *B. burgdorferi s.l.*, *A. phagocytophilum* and *Rickettsia* spp. DNA in tick DNA extracts was tested by PCR using specific primers for each pathogen. The primer sets and PCR conditions used for the different pathogens are listed in Table 1. A *Babesia* spp. specific PCR was based on the amplification of a 411-452 bp fragment of the multicopy 18S rRNA gene [13]. For the detection of *A. phagocytophilum,* a real-time PCR was performed according to [14], to amplify a 77-bp segment at the conserved amino-terminal coding region of the *msp2* gene. All samples, a negative and a positive control were run in duplicate, and samples with a *C*p (crossing point of the amplification curve) above 35 cycles were considered negative. For *Borrelia* spp. a specific nested PCR was developed targeting the flagellin gene, resulting in a 193 bp fragment. The first reaction was carried out in a 25 μl volume containing 0.2 μM of each dNTP, 0.2 μM of each primer, 1.5 mM MgCl_2_, 5 μl of 5× buffer, 0.625 U of Taq and 2.5 μl of the DNA extract. The second reaction was conducted on 2.5 μl of a 1/10 dilution of the first PCR product and contained in 25 μl 0.2 μM of each dNTP, 0.2 μM of each primer, 1.5 mM MgCl_2_, 5 μl of 5× buffer and 0.625 U of Taq. Plasmids containing genomic DNA of *Borrelia* spp. were kindly provided by F. Jongejan (Utrecht University, The Netherlands) and H. Sprong (RIVM, The Netherlands). DNA from *Rickettsia* spp. was detected using the rapidSTRIPE Rickettsia Assay (Analytik Jena, Jena, Germany), following the manufacturer’s instructions. Positive samples were confirmed by PCR, targeting the citrate synthase gene (*gltA)* with the Rsfg877/Rsfg1258 primers, which results in amplification of a 380 bp fragment [15].

**Table 1:** **Primers and polymerase chain reaction conditions for detection of pathogens in *Ixodes, R. sanguineus *and *D. reticulatus***

***Organism detected***	***Target gene***	***Primer sequences (5’-3’)***	***Product t size (bp)***	***Melting temperature***	***Reference***
*Borrelia burgdorferi s.l.*	Flagellin	Outer primers:	236	• Denaturation 95°C 10 min	/
		Bflag2F: GCT GAA GAR CTT GGA ATG CAR CC		• Hybridisation 35 cycles: 95°C 30s, 59°C 30s, 72°C 30s	
		Bflag2R: AGC AGG YGY TGG YTG YTG AGC			
				• Extension 72°C 10 min	
		Inner primers:	193	• Denaturation 95°C 10 min	/
		Bflag2nestF: CWC CAG CRT CAC TTT CAG GR		• Hybridisation 35 cycles: 95°C 30s, 56°C 30s, 72°C 30s	
				• Extension 72°C 10 min	
		Bflag2nestR: GYT GGY TGY TGA GCT CCT TC			
*Anaplasma phagocytophilum*	Major surface protein 2 (*msp2*)	Apmsp2f: ATG GAA GGT AGT GTT GGT TAT GGT ATT	77	• Denaturation 95°C 10 min	Courtney *et al*., 2004
		Apmsp2r TTG GTC TTG AAG CGC TCG TA			
				• Hybridisation 40 cycles: 95°C 15 s, 60°C 1 min	
				• Extension 60°C 1 min	
*Rickettsia* spp.	Citrate synthase (*gltA*)	Rsfg877: GGG GGC CTG CTC ACG GCG G	380	• Denaturation 95°C 10 min	Reis *et al*., 2011
		Rsfg1258: ATT GCA AAA AGT ACA GTG AAC A		• Hybridisation 5 cycles: 95°C 60s, 58°C 60s, 72°C 60s	
				35 cycles: 95°C 60s, 51°C 60s, 72°C 60s	
				• Extension 72°C 10 min	
*Babesia* spp.	18S rRNA	BJ1:GTC TTG TAA TTG GAA TGA TGG	411-452	• Denaturation 94°C 10 min	Casati *et al*, 2006
		BN2:TAG TTT ATG GTT AGG ACT ACG		• Hybridisation: 35 cycles 94°C 1 min, 55°C 1 min, 72° 2 min	
				• Extension 72°C 5 min	

Tick DNA samples positive for *Babesia* spp. were sequenced as described by [13]. Samples that were positive for *A. phagocytophilum* were sent to GATC Biotech AB (Konstanz, Germany) for sequence analysis. *Borrelia* and *Rickettsia* PCR products were purified using the Qiaquick® purification kit (Qiagen) and fully sequenced in both directions using the Big Dye Terminator V3.1 Cycle sequencing Kit (Applied Biosystems). Sequencing reactions were analysed on a 3100 Genetic Analyzer (Applied Biosystems) and assembled with Seqman II (DNASTAR, Madison WI, USA). Sequences were compared with reference sequences by BLAST-analysis against the NCBI database.

### Statistical analysis

Pathogen infection rates and 95% confidence intervals were calculated for each pathogen.

The χ^2^-test was used to compare infection rates of pathogens between *I. ricinus* and *I. hexagonus* ticks, to compare infection rates for the different pathogens in ticks collected from dogs and cats with or without clinical signs indicative for tick-borne disease and to compare tick infection rates between northern Belgium (Flanders) and southern Belgium (Wallonia). A p-value of 0.05 was considered statistically significant.

## Results

### Tick identification

A total of 2373 ticks were collected from 647 dogs and 506 cats. The number of ticks per animal ranged from 1 to 34, but most submissions (70%) only contained a single tick. Most of the collected ticks were *Ixodes* spp*.* In cats, *I. ricinus* (80.1% of infested cats, 95% CI: 77.0–83.9) and *I. hexagonus* (23.4%, 95% CI: 19.9–27.3) were the only tick species found. The most frequently found tick species in dogs was also *I. ricinus* (82.1% of infested dogs, 95% CI: 78.9–84.9), followed by *I. hexagonus* (18.9%, 95% CI: 15.9–22.2). Ticks were submitted throughout the year. Most *I. ricinus* ticks were collected during springtime, with a peak in May 2008 (843 ticks,). Most *I. hexagonus* were also recorded during this period, but a second, smaller peak occurred in October 2008. The majority of the ticks that were collected from dogs, were located on the head (42.7%), the neck (21.7%) and the thorax/abdomen (22.6%). In cats, most ticks were recovered from the head (40.9%) and the neck (41.6%). The majority of the recovered ticks were adults (89.2%), only 8.3% were nymphs and 2.6% larvae.

*R. sanguineus* and *D. reticulatus* were only found on 4 and 6 dogs, respectively. All dogs infested with *R. sanguineus* and 5 out of 6 dogs infested with *D. reticulatus* had a recent travel history (to France, Italy or an unspecified country), but *D. reticulatus* ticks were repeatedly collected from one dog without a history of travelling abroad. The presence of an indigenous population of *D. reticulatus* was confirmed by flagging the area where this dog was regularly taken for a walk (Beveren-Waas, Flanders, 51°12’02”N, 4°14’15”E) (Figure 1) [9].

### Pathogen detection

**Figure 2:**
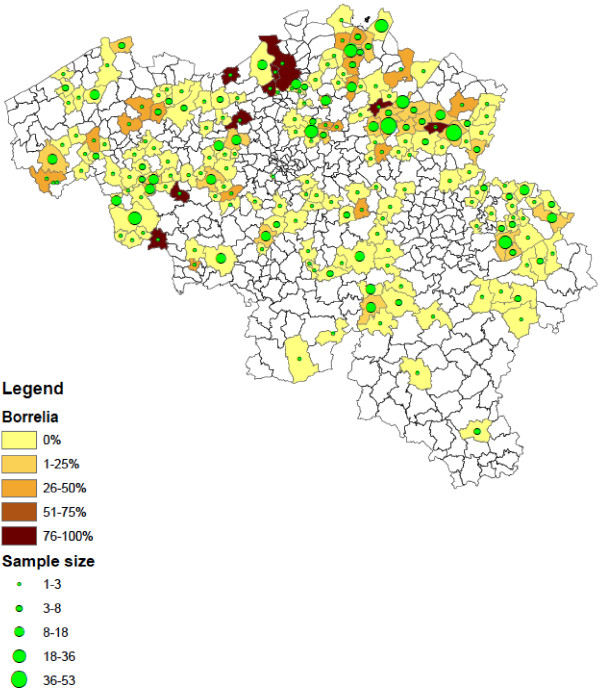
**Distribution of *Ixodes *ticks positive for *Borrelia *spp. in Belgium (infection rates and numbers of ticks submitted per municipality).**

**Figure 3:**
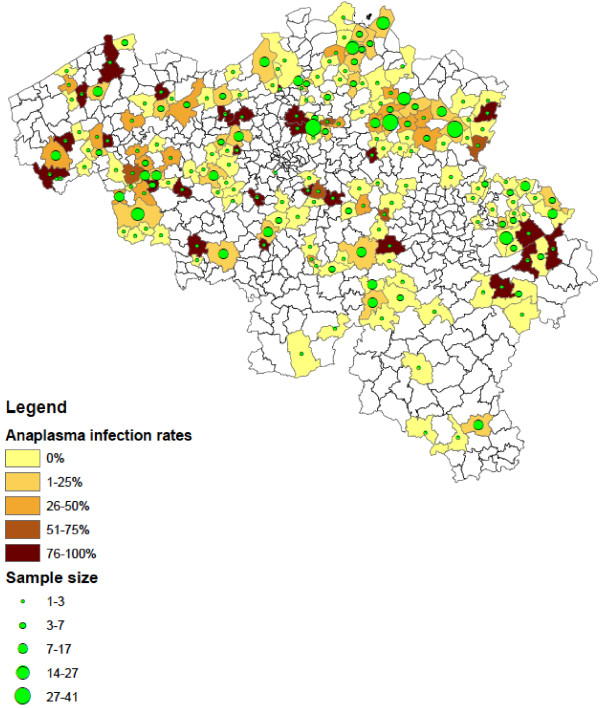
**Distribution of *Ixodes *ticks positive for *A. phagocytophilum *in Belgium (infection rates and numbers of ticks submitted per municipality).**

**Figure 4:**
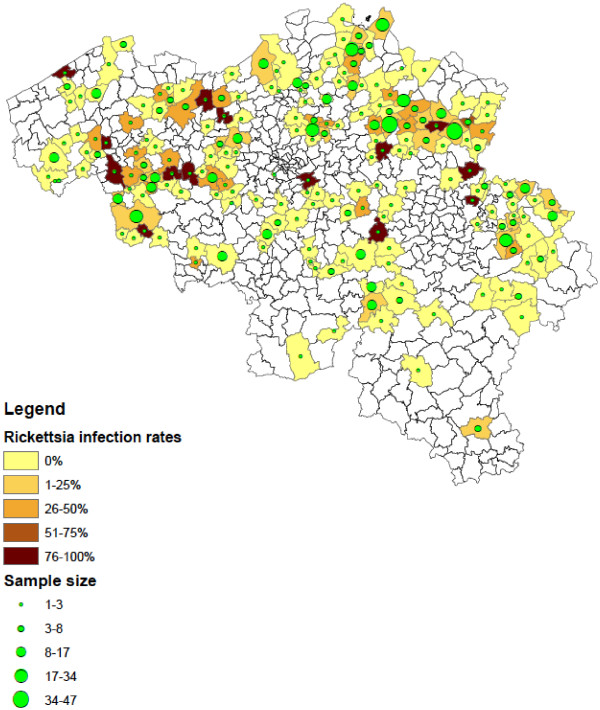
**Distribution of *Ixodes *ticks positive for *R. helvetica *in Belgium (infection rates and numbers of ticks submitted per municipality).**

Of the collected *Ixodes* ticks, 1.3% (95% CI: 0.7–2.3) were found positive for *Babesia* spp*.* These results were described in detail by [13]. In addition, 19.5% (95% CI: 16.9–22.2) *Ixodes* ticks were positive for *A. phagocytophilum* and 10.2% (95% CI: 8.4–12.3) for *Borrelia* spp. *B. afzelii* (4.8%, EMBL accession numbers HF930599 – HF930641)*, B. garinii* (1.8%, EMBL accession numbers HF930648 – HF930672), *B. burgdorferi s.s.* (0.6%, EMBL accession numbers HF930706 – HF930716), *B. valaisiana* (0.5%, EMBL accession numbers HF930642 – HF930647), *B. lusitaniae* (2.1%, EMBL accession numbers HF930682 – HF930705) and *B. spielmanii* (0.7%, EMBL accession numbers HF930673 – HF930681) were detected. *R. helvetica* was found in 14.1% (95% CI: 12.0–16.5) of *Ixodes* ticks (EMBL accession numbers HF930717 – HF930723). Infections with more than one pathogen occurred in 54 *Ixodes* ticks, most of them were infected with 2 pathogens (n = 50). The most common co-infections were *A. phagocytophilum* with *R. helvetica* (n = 18) and *A. phagocytophilum* with *B. afzelii* (n = 11). Ticks positive for the investigated pathogens were detected across the entire country, but significantly more ticks were positive for *Borrelia* (χ2 =19.49, d.f. = 1, P < 0.0001), *A. phagocytophilum* (χ2 =19.68, d.f. = 1, P < 0.0001) and *R. helvetica* (χ2 =20.29, d.f. = 1, P < 0.0001) in the northern part of Belgium (Flanders) compared to the southern part (Wallonia). Figures 2, 3, 4 show the numbers of ticks submitted per municipality and the percentage of ticks positive for *Borrelia* spp. (Figure 2), *A. phagocytophilum* (Figure 3) and *R. helvetica* (Figure 4).

Although *B. burgdorferi s.l.* was detected more frequently in *I. ricinus* (11.1%) than in *I. hexagonus* (6.9%)*,* there was no significant difference between *I. ricinus* and *I. hexagonus* in the proportion of ticks that contained DNA from *B. burgdorferi s.l.* (χ2 =3.19, d.f. = 1, P = 0.07), *A. phagocytophilum* (χ2 =0.40, d.f. = 1, P = 0.52) or *R. helvetica* (χ2 =0.69, d.f. = 1, P = 0.41) (Table 2).

**Table 2:** **Number of *I. ricinus *and *I. hexagonus *ticks positive and negative for *A. phagocytophilum, Borrelia *spp. and *Rickettsia *spp**

	***Ixodes ricinus***	***Ixodes hexagonus***	**Total**
*A. phagocytophilum* pos	127	37	164
*A. phagocytophilum* neg	541	138	679
*Borrelia* pos	83	14	97
*Borrelia* neg	662	190	852
*Rickettsia* pos	100	32	132
*Rickettsia* neg	634	169	803

All *Dermacentor* ticks were negative for all the investigated pathogens, but one *R. sanguineus* tick was found positive for *Rickettsia massiliae* (EMBL accession number HF930724).

Most of the animals were healthy at the time of tick collection. Nevertheless, one cat showed swelling and pain at the site where a *Borrelia-* positive tick was attached. One cat and five dogs that were infested with ticks that contained *A. phagocytophilum* DNA, showed either local swelling or inflammation at the site of tick collection (n = 4, one co-infection with *Borrelia*), lameness (n = 1) or weight loss (n = 1). One dog with a *Rickettsia*-positive tick had arthritis. However, 13 cats and 16 dogs also showed clinical signs that have been associated with tick-borne diseases, such as acute malaise (apathy, fever, anorexia), lameness, lymphadenopathy, glomerulonephritis or neurological signs, while collected ticks were negative for all investigated pathogens. There was no significant difference in the detection rates of *B. burgdorferi s.l.* (χ2 =2.31, d.f. = 1, P = 0.13), *A. phagocytophilum* (χ2 =0.13, d.f. = 1, P = 0.72) or *R. helvetica* (χ2 =3.53, d.f. = 1, P = 0.60) between ticks collected from animals with or without clinical signs.

## Discussion

In this study *I. ricinus* was the predominant tick species infesting companion animals, followed by the hedgehog tick, *I. hexagonus*. This is in analogy with other studies in North-Western Europe. Similar results were reported from The Netherlands [3], Germany [16] and the UK [17,18]. In the UK and Ireland, the fox tick *I. canisuga* was also frequently recovered from dogs [17,18]. Ogden *et al*. [17] found *I. ricinus* on a significantly higher proportion of dogs than cats*,* while *I. hexagonus* was more frequently found on cats. They suggested that differences in behaviour between dogs and cats could affect their likelihood of encountering both *Ixodes* species. This hypothesis was not supported by our data, with similar proportions of dogs and cats carrying *I. ricinus* and *I. hexagonus.* Equal proportions of both *Ixodes* species were also recovered from dogs and cats in Germany [16] and The Netherlands [3]. The recovered *Ixodes* ticks were mainly adults, as was also observed in other studies in dogs and cats [16,18,19]. Although it cannot be excluded that some nymphs and larvae were overlooked in the clinical inspections, adult *Ixodes* ticks are known to attach preferably to large or medium-sized mammals, including dogs and cats [20].

Generally, nymphs and adults of *I. ricinus* show a marked seasonal variability in their questing activity, with a first peak in late spring and early summer and a second peak in autumn [21,22]. This seasonal pattern was also observed in the number of ticks recovered from dogs by [18] and [19]. In this study, we observed an obvious spring peak, but only low numbers of *I. ricinus* were submitted in autumn. Although participation rates of the veterinarians may have introduced a bias, tick questing activity may be variable from year to year and a single spring or summer peak has previously been observed for questing *I. ricinus* nymphs [23] and for adult *I. ricinus* on dogs [16]. Most *I. hexagonus* were also recovered in the spring in our study, but a second, smaller peak occurred in October. Only limited published information is available on the seasonal abundance of *I. hexagonus* ticks, but seasonal fluctuations in numbers of *I. hexagonus* collected from dogs and hedgehogs are generally weaker compared to *I. ricinus*[16,24].

The majority of infected ticks were found in Northern Belgium (Flanders). The north-eastern part of Belgium (the Campine) is known to be heavily infested with *Ixodes* and has a relatively high incidence of Lyme borreliosis [25]. Although the south-eastern part of Belgium has a lot of forest and would also be expected to have a lot of good *Ixodes* habitats, the number of ticks positive for tick-borne diseases from that area was lower in our study. The reason for this remains unclear.

There was no significant difference between *I. ricinus* and *I. hexagonus* in the proportion of ticks that contained DNA from *B. burgdorferi s.l.*, *A. phagocytophilum* or *R. helvetica*. Although both *I. ricinus* and *I. hexagonus* can be vectors of *Borrelia* spp. and *A. phagocytophilum*[26,27], *I. ricinus* is considered to be the principal vector of these pathogens [27,28]. As a nest dwelling species, *I. hexagonus* will have little direct contact with humans. Nevertheless, *I. hexagonus* could be a vector for the transmission of *Borrelia* spp. and *A. phagocytophilum* to hedgehogs, and then via co-infections with *I. ricinus* indirectly to humans [24,27].

The percentage of ticks positive for *Borrelia* spp. was within the range of infection rates (2.3-22%) in ticks collected from dogs in other European countries [3,16,29-31]. Similar infection rates were also reported in questing *I. ricinus* in Belgium (12-23%) [32,33]. Within the *B. burgdorferi sensu lato* group, *B. afzelii* and *B. garinii* are the most common species in The Netherlands, Belgium and northern France, while *B. burgdorferi s.s.* is less common in this region [34]. *B. valaisiana* has also been repeatedly found in The Netherlands (*e.g.* 3, 29). Together with other recent studies in Belgium and Luxembourg [33,35] our results show that besides *B. valaisiana, B. spielmanii* and *B. lusitaniae* are also present in *Ixodes* in the Benelux region. *B. garinii, B. afzelii* and *B. burgdorferi s.s.* are well known to be pathogenic for humans, but the pathogenic significance of the other species is still unclear [36,37].

The majority of dogs and cats that are exposed to *Borrelia* infections remain clinically normal [20], which was also the case in this study. Most cases of canine Lyme borreliosis are associated with *B. burgdorferi s.s.* Whilst *B. burgdorferi s.s.* is the only *Borrelia* species in the USA, prevalences of *B. burgdorferi s.s.* in Europe are much lower [34], as was also the case in this study. This may explain why lyme borreliosis is frequently diagnosed in dogs in endemic regions in the USA, but less frequently in European dogs.

Although *Borrelia* can be transmitted from dogs to ticks*,* dogs are not considered as important reservoir hosts [20] and studies examining seropositivity in dogs, their owners and other local residents found no correlation between dog ownership and infection risk [38-40]. Nevertheless, dogs and cats can be used as sentinels for lyme borreliosis. Serological studies in the USA showed that exposure of dogs to *B. burgdorferi* mimics the geographical distribution of reports of Lyme borreliosis in humans [20] and the use of dog sera to detect and quantify the risk of Lyme borreliosis for humans in a certain region is considered to be more sensitive than the use of incidence reports of human clinical cases [40]. Seroprevalence has been found greater in dogs than in humans due to their greater habitat exposure, lack of protective clothing and inability to check themselves from ticks [38]. The use of dog sera also has the advantage over human serology that the seroprevalence among dogs is more likely to reflect the actual environmental risk of Lyme borreliosis, because of the short half-life of canine antibodies against *B. burgdorferi*[40]. However, serological studies are often limited by small sample sizes, and false positive results are possible. Detection of *Borrelia* DNA in ticks collected from dogs and cats can be a valuable alternative [31].

The *A. phagocytophilum* infection rate in this study was much higher than previously reported point prevalences in *Ixodes* ticks from dogs in The Netherlands and Poland (1.6-2.9%) [3,30]. Seroprevalences in dogs in Europe are also very variable, with prevalences from < 5% to > 50% [41]. The percentage of seropositive dogs depends on the dog population sampled (*e.g.* healthy dogs vs. dogs with signs of tick-borne diseases) and geographical variation in exposure to ticks and reservoir hosts. The high infection rate of *A. phagocytophilum* in ticks collected from dogs in this study is in contrast with the low incidence of human granulocytic anaplasmosis (HGA) in Belgium (< 100/year) [25]. However, there is a discrepancy between the official (low) incidence rates [25] and the high number of HGA cases that are detected in specific surveys [42,43]. In a recent 10-year serological survey in patients with symptoms of tick-borne infections, 31% of the samples were positive, and 111 cases of HGA were confirmed [42]. These data suggest that Belgium is a hot spot for HGA and that many cases of HGA probably remain undiagnosed [43].

Most dogs infected with *A. phagocytophilum* probably remain healthy [41]. The most common clinical signs in dogs that develop illness are lethargy, fever and lameness. In the present study, no association was found between clinical signs and the presence of *A. phagocytophilum* in ticks collected from these animals. Although identical 16S rRNA gene sequences have been found in canine and human isolates of *A. phagocytophilum* in Europe [44,45], dogs are not thought to be important reservoirs for *A. phagocytophilum*, since bacteremia is of short duration in this species [46]. In Slovenia, no difference in seroprevalence was observed between people with or without exposure to dogs [47].

*Ixodes* spp. can transmit several *Rickettsia* species belonging to the ‘spotted fever’ group, such as *Rickettsia helvetica* and *R. monacensis*. We found *R. helvetica* DNA in 14.1% of *Ixodes* ticks collected from dogs and cats in Belgium. In the Netherlands, 24.7% *I. ricinus* ticks and 0.8% *I. hexagonus* ticks collected from dogs, cats and a hedgehog were infected with *R. helvetica*[3]. In Switzerland, 40.9% and 17.6% of *Ixodes* ticks collected from cats and dogs, respectively, tested positive for *R. helvetica* with a *gltA*-specific TaqMan PCR system [48]. *R. helvetica* is a suspected pathogen in humans. Symptoms that have been associated with *R. helvetica* infections include fever, headache, arthralgia, myalgia and perimyocarditis [48]. The high prevalence of *R. helvetica* in *Ixodes* spp. and the high abundance of these tick species suggest that the likelihood of transmission of *R. helvetica* to humans should be high [48]. However, despite a high infection rate (19%) of *R. helvetica* in ticks collected from humans in the Netherlands, no association between symptoms and *R. helvetica* was found [49].

The clinical importance of *R. helvetica* in domestic animals is as yet uncertain and it is also unknown whether dogs and cats can serve as a reservoir after infection. The fact that the estimated prevalence of *R. helvetica* in ticks collected from dogs, cats and roe deer was higher than in ticks collected from the vegetation [48,50] may indicate that large animals act as a reservoir for *R. helvetica*[48].

Next to *I. ricinus* and *I. hexagonus,* small numbers of *R. sanguineus* and *D. reticulatus* were collected from dogs. All submitted *R. sanguineus* ticks were considered to be imported, since they were all collected from dogs with a history of travelling abroad, mostly to Southern Europe. One *R. sanguineus* tick contained DNA from *R. massiliae. R. massiliae* is suspected to be the main cause of Mediterranean spotted fever in Spain [51].

Although most submitted *Dermacentor* ticks were also from dogs with a travel history, *D. reticulatus* ticks were repeatedly sampled from one particular dog that had never been outside Belgium. Flagging confirmed the presence of questing *Dermacentor* ticks in the area where the dog was walked daily [9]. This was the first finding of an indigenous population of *D. reticulatus* in Belgium. Further investigations have revealed the presence of at least 4 other foci of *D. reticulatus* in Belgium (51, M. Madder, unpublished results).

## Conclusions

Repeated collection of *D. reticulatus* from a dog without history of traveling led to the discovery of an indigenous population of *D. reticulatus* in Belgium [9], confirming the geographical expansion of this tick species in North-western Europe. High infection rates were found for *Borrelia* spp., *A. phagocytophilum* and *R. helvetica* in *Ixodes* ticks collected from dogs and cats in Belgium. *B. lusitaniae* and *R. helvetica* were detected in ticks in Belgium for the first time.

## Abbreviations

TBEV: Tick-borne encephalitis virus.

## Competing interests

This study was partially financed by Bayer Health Care, Animal Health Division. In the last 5 years, EC has obtained funding from, lectured or consulted for the following companies selling products to treat tick infestations in dogs and cats: Bayer Health Care, Merial, Pfizer Animal Health.

## Authors’ contributions

EC conceived of and designed the study, acquired funding, flagged for *D. reticulatus* in Beveren-Waas, conducted data analysis and drafted the manuscript. BL conceived of and designed the study, acquired funding and helped drafting the manuscript. CC and PH performed the real-time PCR analyses for *A. phagocytophilum* and flagged for *D. reticulatus* in Beveren-Waas*.* SC developed and performed the PCR analyses for *Borrelia,* carried out the rapid assay and PCRs for *Rickettsia* and conducted data analysis. A-CD participated in the design of the study and helped drafting the manuscript. ADC contributed to the organisation of the study and identified the ticks. MM helped drafting the manuscript and flagged for *D. reticulatus* in Beveren-Waas. CS helped in the design of the study. LL contributed to the organisation of the study, identified the ticks, developed and performed the *Babesia* PCR, conducted data analysis and helped drafting the manuscript. All authors read and approved the final manuscript.
